# Lung Donation and Transplant Recipient Outcomes at Independent vs Hospital-Based Donor Care Units

**DOI:** 10.1001/jamanetworkopen.2024.17107

**Published:** 2024-06-25

**Authors:** Emily A. Vail, Xingmei Wang, Douglas E. Schaubel, Peter P. Reese, Edward Cantu, Niels D. Martin, Peter L. Abt, Kim M. Olthoff, Meeta P. Kerlin, Jason D. Christie, Mark D. Neuman

**Affiliations:** 1 Department of Anesthesiology & Critical Care, University of Pennsylvania Perelman School of Medicine, Philadelphia; 2 Penn Center for Perioperative Outcomes Research and Transformation, Philadelphia, Pennsylvania; 3 Leonard Davis Institute of Health Economics, Philadelphia, Pennsylvania; 4 Department of Biostatistics, Epidemiology, and Informatics, University of Pennsylvania Perelman School of Medicine, Philadelphia; 5 Renal-Electrolyte and Hypertension Division, Department of Medicine, Hospital of the University of Pennsylvania, Philadelphia; 6 Penn Transplant Institute, Philadelphia, Pennsylvania; 7 Division of Cardiothoracic Surgery, Department of Surgery, Hospital of the University of Pennsylvania, Philadelphia; 8 Department of Surgery, Hospital of the University of Pennsylvania, Philadelphia; 9 Division of Pulmonary, Allergy and Critical Care Medicine, Department of Medicine, Hospital of the University of Pennsylvania, Philadelphia

## Abstract

**Importance:**

Centralizing deceased organ donor management and organ recovery into donor care units (DCUs) may mitigate the critical organ shortage by positively impacting donation and recipient outcomes.

**Objective:**

To compare donation and lung transplant outcomes between 2 common DCU models: independent (outside of acute-care hospitals) and hospital-based.

**Design, Setting, and Participants:**

This is a retrospective cohort study of Organ Procurement and Transplantation Network deceased donor registry and lung transplant recipient files from 21 US donor service areas with an operating DCU. Characteristics and lung donation rates among deceased donors cared for in independent vs hospital-based DCUs were compared. Eligible participants included deceased organ donors (aged 16 years and older) after brain death, who underwent organ recovery procedures between April 26, 2017, and June 30, 2022, and patients who received lung transplants from those donors. Data analysis was conducted from May 2023 to March 2024.

**Exposure:**

Organ recovery in an independent DCU (vs hospital-based DCU).

**Main Outcome and Measures:**

The primary outcome was duration of transplanted lung survival (through December 31, 2023) among recipients of lung(s) transplanted from cohort donors. A Cox proportional hazards model stratified by transplant year and program, adjusting for donor and recipient characteristics was used to compare graft survival.

**Results:**

Of 10 856 donors in the starting sample (mean [SD] age, 42.8 [15.2] years; 6625 male [61.0%] and 4231 female [39.0%]), 5149 (primary comparison group) underwent recovery procedures in DCUs including 1466 (28.4%) in 11 hospital-based DCUs and 3683 (71.5%) in 10 independent DCUs. Unadjusted lung donation rates were higher in DCUs than local hospitals, but lower in hospital-based vs independent DCUs (418 donors [28.5%] vs 1233 donors [33.5%]; *P* < .001). Among 1657 transplant recipients, 1250 (74.5%) received lung(s) from independent DCUs. Median (range) duration of follow-up after transplant was 734 (0-2292) days. Grafts recovered from independent DCUs had shorter restricted mean (SE) survival times than grafts from hospital-based DCUs (1548 [27] days vs 1665 [50] days; *P* = .04). After adjustment, graft failure remained higher among lungs recovered from independent DCUs than hospital-based DCUs (hazard ratio, 1.85; 95% CI, 1.28-2.65).

**Conclusions and Relevance:**

In this retrospective analysis of national donor and transplant recipient data, although lung donation rates were higher from deceased organ donors after brain death cared for in independent DCUs, lungs recovered from donors in hospital-based DCUs survived longer. These findings suggest that further work is necessary to understand which factors (eg, donor transfer, management, or lung evaluation and acceptance practices) differ between DCU models and may contribute to these differences.

## Key Points

**Question:**

Does the survival of recipients with lungs recovered and transplanted from deceased donors after brain death differ between independent and hospital-based donor care units?

**Findings:**

In this cohort study of 10 856 donors and 1657 recipients, although lung donation rates were higher among donors cared for in independent donor care units, graft survival was longer among donor lungs recovered from hospital-based units.

**Meaning:**

These findings suggest that differences in lung donation and transplantation outcomes exist and depend on the type of donor care unit used for deceased organ donor management and organ recovery.

## Teaser

This cohort study compares lung donation rates and transplant survival outcomes in independent vs hospital-based donor care units (DCUs).

## Introduction

Centralization of deceased organ donors after brain death into donor care units (DCU) addresses practical challenges associated with hospital-based management^1^ and is proposed to address the critical organ shortage in the US.^2^ DCUs are operated by organ procurement organizations (OPOs)—federal contractors responsible for organ donor identification, authorization, and organ allocation in discrete regions. Nearly one-half of OPOs operate a DCU in 1 of 2 commonly used models: (1) hospital-based, sharing some or all resources (including staff and beds) with hospitalized patients (under contract with the hospital), and (2) independent, which resemble intensive care units and operating rooms but lack hospital affiliations or licensed beds.^3^

While individual OPOs operate DCUs according to available local hospital resources, operational needs, and finances, each DCU model offers unique advantages, which include lower donor management costs among donors in independent DCUs,^4^ and access to consultant physicians or additional clinical resources (eg, cardiac catheterization laboratories) in hospital-based DCUs.^3^ Differences between models may be particularly impactful for lung donation because some acute complications of brain death (eg, atelectasis) are mitigated with lung donor–specific protocols^5^ or interventions (eg, lung-protective ventilation^6^ or prone positioning^7^), which may be delivered more consistently in independent DCUs than hospitals.^8^

Higher rates of lung donation are reported among donors cared for in independent DCUs,^8,9^ but recipient survival—a patient-centered measure and primary metric of lung transplant program performance^10^—has not been examined. Therefore, this study aims to compare graft survival duration between lungs recovered from deceased organ donors after brain death in each DCU model. Because many factors impact survival after transplant, we hypothesized that graft survival duration would not differ between lungs recovered from donors in hospital-based vs independent DCUs.

## Methods

### Study Design

We conducted a retrospective cohort study using preexisting data. The study protocol was determined exempt from human participants research and the requirement for informed consent by the Penn Medicine institutional review board and follows the Strengthening the Reporting of Observational Studies in Epidemiology (STROBE) reporting guideline for observational studies.^11^ This study used data from the Organ Procurement and Transplantation Network (OPTN). The OPTN data system includes data on all donors, wait-listed candidates, and transplant recipients in the US, submitted by members of the OPTN. The Health Resources and Services Administration of the US Department of Health and Human Services provides oversight to the activities of the OPTN contractor. Analyses reflect data captured in study datasets as of February 4, 2024.

### Study Sample

The study included all deceased donors captured in the study dataset who underwent recovery procedures in the US between April 26, 2017 (the first day recovery location data were collected), and June 30, 2022 (the latest data available at study initiation), and recipients of transplanted lungs from those donors. We excluded donors cared for by OPOs without an operating DCU or with a newly opened DCU (contributing fewer than 6 months of data). We excluded donors unlikely to be transferred to a DCU, including those younger than 16 years and donors after circulatory death. We also excluded donors with recovery procedures before local DCU opening and missing or ambiguous recovery procedure locations.

### Primary Exposure

The primary exposure was the type of DCU where the organ recovery procedure occurred, categorized as hospital-based or independent. Organ donors were classified using recovery procedure location and name variables in the study dataset as previously described,^9^ then categorized according to published literature.^3^ Recipients of lungs from cohort donors were also compared according to the site of lung recovery.

Most donor and recipient characteristics, including sex, race and ethnicity, donation outcomes, recipient severity of illness measures (including lung allocation score at the time of transplant^12^), transplant characteristics (eg, waitlist duration or single vs double transplant), and transplantation outcomes were defined by OPTN. Race and ethnicity were recorded by organ transplant coordinators and transplant program staff at the time of organ donor assessment and patient waitlist registration, respectively.^13^ Race and ethnicity categories included American Indian or Alaska Native, Asian, Black, Hispanic, Native Hawaiian or Other Pacific Islander, White, and multiracial. The study assessed these characteristics because of known differences in rates of deceased organ donation^14^ and lung transplant access and outcomes between groups.^15,16^ Recipient diagnoses were classified according to Valapour et al.^17^ We determined some transplant factors using combinations of individual donor and recipient characteristics, including extended criteria for lung donation^18^ and height mismatch.^19^

### Study Outcomes

The primary outcome was the duration of transplanted lung survival (as of December 31, 2023, with graft failure defined as patient death or retransplant). Donation process outcomes included donor management time (from brain death diagnosis to aortic cross-clamp application) and total ischemic times. Organ donation outcomes included the donation of at least 1 transplanted lung and the number of lungs and other organs transplanted from each donor. We also examined secondary recipient outcomes 72 hours after transplant (including receipt of mechanical ventilation), at hospital discharge (including length of stay after transplant), and the incidence of graft survival at 1 year.

### Statistical Analysis

We performed unadjusted comparisons of the demographic and clinical characteristics of cohort donors cared for in hospital-based vs independent DCUs. Then, we repeated these comparisons in the subgroup of donors who donated at least 1 lung for transplant. Next, we compared the characteristics and outcomes of patients who received lung transplants from cohort donors who underwent organ recovery in each DCU type. Other than organ recovery location data (excluded during cohort selection), missing data were minimal. Variables missing more than 10% of recorded values were not considered in analyses.

To examine the duration of graft survival (the primary outcome), we excluded recipients of simultaneous multiorgan transplants. We created Kaplan-Meier curves and compared restricted mean survival times between lungs recovered from donors in each DCU type over the first 5 years of follow-up.^20^ To account for differences in donor and transplant recipient characteristics likely to affect graft survival duration, we created a Cox proportional hazards model stratified by transplant year and transplant program (to account for changes in allocation policy that may have changed lung utilization practices and differences in transplant center characteristics associated with recipient outcomes). We adjusted for clinically relevant donor and recipient factors associated with primary graft dysfunction and short- and long-term recipient survival (see the eFigure in Supplement 1 for a proposed causal diagram).^12,17,18,19,21,22,23^ From the Cox model, we also estimated the average adjusted 1-year graft survival in each group. We tested the proportionality assumption in the adjusted model as described in the eMethods in Supplement 1.

Analyses were conducted using SAS 9.4 (SAS Institute). We considered a 2-sided *P* < .05 as the threshold of statistical significance. Data analysis occurred from May 2023 to March 2024.

## Results

**Figure 1.**
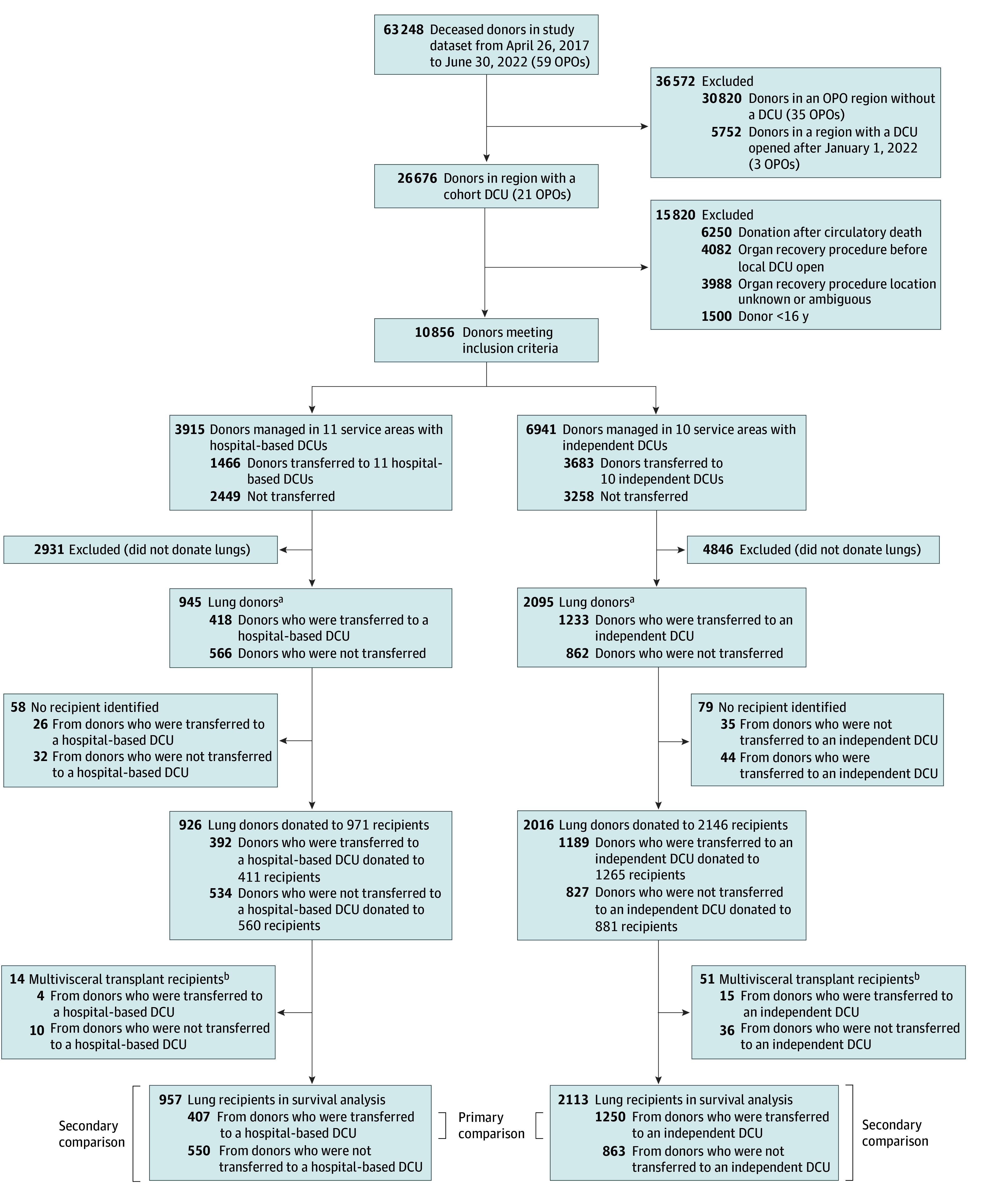
Cohort Selection Diagram Secondary analyses compared donors in hospitals in donor service areas with operating DCUs but not transferred to a DCU (stratified by DCU type) and are presented in eTables 1-3 in Supplement 1. DCU indicates donor care unit; OPO, organ procurement organization. ^a^Donated at least 1 lung for transplant. ^b^Recipient received at least 1 donated lung from a cohort donor combined with 1 or more additional solid organs.

The study dataset captured 63 248 deceased donors after brain death, of which 10 856 (mean [SD] age, 42.8 [15.2] years; 6625 male [61.0%] and 4231 female [39.0%]) met inclusion criteria and were cared for by OPOs with an operating DCU. Of these, 5149 donors (47.4%) underwent recovery procedures in DCUs (Figure 1). A majority of these donors underwent recovery in 1 of 10 independent DCUs (3683 donors [71.5%]); the remainder (1466 donors [28.4%]) underwent recovery in 1 of 11 hospital-based DCUs (eTable 1 in Supplement 1). Among these 5149 donors, 1651 (32.1%) donated at least 1 lung for transplant; rates of lung donation (and the number of lungs transplanted) were higher among donors cared for in independent vs hospital-based DCUs (1233 donors [33.5%] vs 418 donors [28.5%]; *P* < .001), but higher in each DCU type than local hospitals (eTable 2 in Supplement 1).

Compared with lung donors in hospital-based DCUs, those in independent DCUs were less likely to be Hispanic (162 of 1233 donors [13.1%] vs 93 of 418 donors [22.2%]) and more likely to be White (804 of 1233 donors [65.2%] vs 216 of 418 donors [51.7%]) (Table 1). Intracranial hemorrhage or stroke was the most common cause of death in both groups. Lung donors who underwent recovery in independent DCUs were significantly less likely to have documented pulmonary infection during donor management (838 of 1233 donors [68.0%] vs 325 of 418 donors [77.8%]; *P* < .001). Median terminal values for the ratio of partial pressure of oxygen in arterial blood to the fraction of inspiratory oxygen concentration (Pao_2_:Fio_2_) were similar between groups.

**Table 1.** Characteristics of Cohort Donors That Donated at Least 1 Lung for Transplant

Characteristic	Donors, No. (%) (N = 1651)^a^		*P* value
	Hospital-based DCU (n =418)	Independent DCU (n = 1233)	
DCUs, No./Total No.	11/21 (52.4)	10/21 (47.6)	NA
Donation year, No./Total No. (%)			
April to December 2017	28/102 (27.4)	74/102 (72.5)	.02
2018	39/204 (19.1)	165/204 (80.9)	
2019	47/248 (19.0)	201/248 (81.0)	
2020	92/340 (27.1)	248/340 (72.9)	
2021	125/462 (27.1)	337/462 (72.9)	
January to June 2022	87/295 (29.5)	208/295 (70.5)	
Age, mean (SD), y	36.1 (13.1)	36.3 (12.8)	.80
Sex^b^			
Female	163 (39.0)	490 (39.7)	.79
Male	255 (61.0)	743 (60.3)	
Race and ethnicity^b^			
American Indian or Alaska Native	2 (0.5)	5 (0.4)	<.001
Asian	23 (5.5)	11 (0.9)	
Black	78 (18.7)	245 (19.9)	
Hispanic	93 (22.2)	162 (13.1)	
Multiracial	5 (1.2)	5 (0.4)	
Native Hawaiian or Other Pacific Islander	1 (0.2)	1 (0.1)	
White	216 (51.7)	804 (65.2)	
History of tobacco use	29 (6.9)	118 (9.6)	.26
History of cocaine use	69 (16.5)	258 (20.9)	.03
History of other drug use	225 (53.8)	697 (56.5)	.62
Clinical characteristics			
Donor height, median (IQR), cm	172 (163-179)	173 (165-180)	.15
Body mass index, median (IQR)^c^	25.8 (22.5-29.6)	26.3 (22.9-30.3)	.003
Mechanism of death			
Asphyxiation	32 (7.7)	53 (4.3)	.03
Blunt injury	84 (20.1)	233 (18.9)	
Cardiovascular	38 (9.1)	125 (10.1)	
Death from natural causes	8 (1.9)	28 (2.3)	
Drowning	2 (0.5)	6 (0.5)	
Drug intoxication	59 (14.1)	255 (20.7)	
Electrical	0	2 (0.2)	
Gunshot wound	85 (20.3)	204 (16.5)	
Intracranial hemorrhage or stroke	105 (25.1)	295 (23.9)	
Other	1 (0.2)	9 (0.7)	
Seizure	4 (1.0)	21 (1.7)	
Stab	0	2 (0.2)	
Terminal Pao_2_:Fio_2_ ratio, mm Hg, median (IQR)	439 (360-507)	447 (361-502)	.53
Pulmonary infection	325 (77.8)	838 (68.0)	<.001

Abbreviations: DCU, donor care unit; NA, not applicable; Pao_2_:Fio_2_, ratio of partial pressure of oxygen in arterial blood to the fraction of inspiratory oxygen concentration.

^a^ Not all cohort donors donated lungs to a lung transplant recipient included in survival analysis (see cohort selection diagram).

^b^ As classified by the Organ Procurement and Transplantation Network.^13^

^c^ Body mass index was calculated as weight in kilograms divided by height in meters squared.

Of 1657 recipients of lungs recovered from donors in DCUs (mean [SD] age, 58.6 [11.9] years; 1068 male [64.4%] and 589 female [35.6%]), 1250 (75.4%) received lungs recovered from independent DCUs and 407 (24.6%) received lungs from hospital-based DCUs (Table 2). Compared with recipients of lungs from hospital-based DCUs, recipients from independent DCUs were similar in age and sex but were less likely to be Asian or Hispanic. Recipients of lungs recovered from independent DCUs had several characteristics associated with better survival after transplant, including higher rates of chronic obstructive pulmonary disease (339 of 1250 recipients in independent DCUs [27.1%] vs 88 of 407 recipients in hospital-based DCUs [21.6%]) and lower rates of restrictive lung disease (779 of 1250 recipients in independent DCUs [62.3%] vs 289 of 407 in hospital-based DCUs [71.0%]). Compared with recipients of lungs from hospital-based DCUs, recipients of lungs from independent DCUs also had significantly longer median (IQR) 6-minute walk distances before transplant (767 [400-1050] ft vs 668 [295-1017] ft; *P* = .04) and lower median (IQR) lung allocation scores at transplant match (37.9 [34.0-45.7] vs 39.0 [34.7-46.8]; *P* = .02). Most transplant characteristics (including double lung transplant and total ischemic times) were similar between recipients of lungs from each DCU type. Cytomegalovirus mismatch rates were also lower among lung recipients from independent DCUs.

**Table 2.** Cohort Transplant Recipient Characteristics by Lung Recovery Location

Characteristic	Transplant recipients, No. (%) (N = 1657)^a^		*P* value
	Hospital-based DCU (n = 407)	Independent DCU (n = 1250)	
Transplant programs, No./Total No.^b^	56/71 (78.9)	66/71 (92.3)	NA
Demographic characteristics			
Transplant year, No./Total No. (%)			
April to December 2017	26/99 (26.3)	73/99 (73.7)	.001
2018	39/212 (18.4)	173/212 (81.6)	
2019	43/247 (17.4)	204/247 (82.5)	
2020	87/347 (25.1)	260/347 (74.9)	
2021	128/463 (27.6)	335/463 (72.4)	
January to June 2022	84/289 (29.1)	205/289 (70.9)	
Age, mean (SD), y	59.5 (11.1)	58.3 (12.1)	.07
Sex^c^			
Female	138 (33.9)	451 (36.1)	.43
Male	269 (66.1)	799 (63.9)	
Race and ethnicity^c^			
American Indian or Alaska Native	6 (1.5)	4 (0.3)	.003
Asian	23 (5.7)	33 (2.6)	
Black	37 (9.1)	109 (8.7)	
Hispanic	62 (15.2)	147 (11.8)	
Multiracial	4 (1.0)	4 (0.3)	
Native Hawaiian or Other Pacific Islander	2 (0.5)	2 (0.2)	
White	273 (67.1)	951 (76.1)	
Clinical characteristics			
Height, median (IQR), cm	170 (163-178)	170 (163-178)	.30
Body mass index, median (IQR)^d^	26.4 (23.5-29.0)	26.2 (22.7-29.3)	.46
Diagnosis group^e^			
A. Obstructive	88 (21.6)	339 (27.1)	.01
B. Pulmonary vascular	19 (4.7)	72 (5.8)	
C. Cystic fibrosis and immunodeficiency	11 (2.7)	60 (4.8)	
D. Restrictive	289 (71.0)	779 (62.3)	
Hospitalized before transplant			
Hospitalized (ICU)	76 (18.7)	180 (14.4)	.06
Hospitalized (non–ICU)	49 (12.0)	131 (10.5)	
Not hospitalized	282 (69.3)	939 (75.1)	
Systolic pulmonary artery pressure, mm Hg, median (IQR)	38 (30-49)	38 (32-48)	.69
Serum creatinine, median (IQR), mg/dL	0.8 (0.7-1.0)	0.8 (0.7-1.0)	.07
Serum bilirubin, median (IQR), mg/dL	0.5 (0.3-0.7)	0.5 (0.3-0.6)	.16
6-min Walk distance, median (IQR), ft	688 (295-1017)	767 (400-1050)	.04
Unable to ambulate	31 (7.6)	123 (9.8)	.18
Panel reactive antibodies at time of transplant, median (IQR), No.	0 (0-7)	0 (0-3)	.31
Mechanical ventilation at time of transplant	29 (7.1)	81 (6.5)	.65
Extracorporeal membrane oxygenation at time of transplant	38 (9.3)	99 (7.9)	.37
Time on waitlist, median (range), d	41 (15-122)	37 (13-97)	.38
Lung allocation score at transplant, median (IQR)	39.0 (34.7-46.8)	37.9 (34.0-45.7)	.02
Transplant characteristics			
Type of transplant			
Single	81 (19.9)	283 (22.6)	.25
Double	326 (80.1)	967 (77.4)	
Retransplant^f^	9 (2.2)	10 (0.8)	.02
Total ischemic time, median (IQR), h	5.5 (4.6-6.9)	5.4 (4.4-6.6)	.16
Extended criteria lung donor^g^	174 (42.8)	548 (43.8)	.70
Height mismatch (donor height vs recipient height)			
>15 cm Taller	29 (7.1)	109 (8.7)	.53
10-15 cm Taller	41 (10.1)	141 (11.3)	
5-10 cm Taller	64 (15.7)	224 (17.9)	
Within 5 cm	164 (40.3)	445 (35.6)	
5-10 cm Shorter	57 (14.0)	189 (15.1)	
10-15 cm Shorter	30 (7.4)	88 (7.0)	
>15 cm Shorter	22 (5.4)	54 (4.3)	
Cytomegalovirus mismatch			
Donor positive and recipient negative	110 (27.0)	318 (25.4)	.002
Donor and recipient positive	183 (45.0)	485 (38.8)	
Donor negative	111 (27.3)	446 (35.7)	

Abbreviations: DCU, donor care unit; ICU, intensive care unit; NA, not applicable.

SI conversion factors: To convert serum bilirubin to micromoles per liter, multiply by 17.104; serum creatinine to micromoles per liter, multiply by 88.4.

^a^ Included in graft survival analyses.

^b^ Some recipients’ transplant programs accepted donor lungs from both DCU types.

^c^ As classified by the Organ Procurement and Transplantation Network.^13^

^d^ Calculated as weight in kilograms divided by height in meters squared.

^e^ Classified according to Valapour et al.^17^

^f^ Recipient with a history of at least 1 lung transplant before the study period.

^g^ As defined by Christie et al.^18^

In survival analyses of recipients of transplanted lungs recovered from DCUs, the median (range) duration of follow-up was 734 (0-2292) days among all grafts and 762 (96-2292) days among those with censored outcomes. Among cohort recipients of lungs from DCUs, there were 451 patient deaths and 19 retransplants; 6 recipients were lost to follow-up during the study period (all among recipients of lungs from independent DCUs). The overall incidence of graft loss (death or retransplant) was higher among recipients of lungs recovered in independent DCUs than hospital-based DCUs (377 recipients [30.2%] vs 93 recipients [22.9%]; *P* = .005).

**Figure 2.**
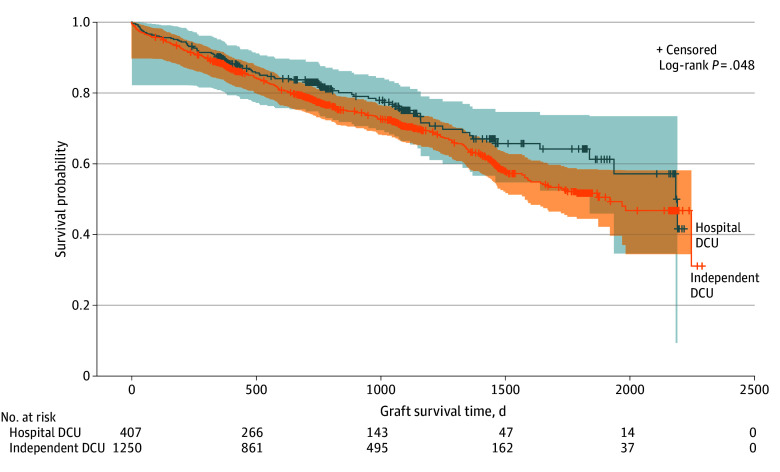
Unadjusted Graft Survival Between Donors in Hospital-Based vs Independent Donor Care Units (DCUs) The figure shows lung transplant recipients with lung(s) recovered in hospital-based DCUs and independent DCUs. Shading represents 95% CIs for the estimates. Crosses indicate censoring.

**Figure 3.**
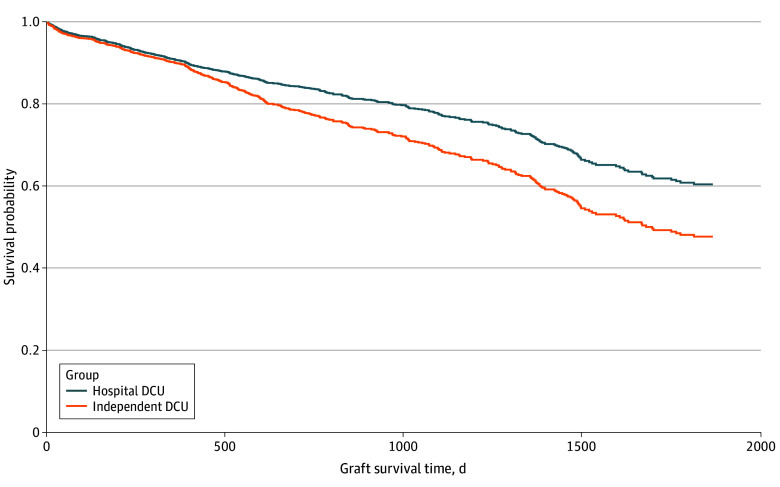
Adjusted Probability of Graft Survival From Hospital-Based vs Independent Donor Care Units (DCUs) The figure shows estimated adjusted graft survival probability among recipients of lungs recovered from hospital-based DCUs and independent DCUs over time. Curves were adjusted for transplant year, transplant program, extended criteria lung donor status, recipient age, sex, race and ethnicity, lung allocation score at the time of transplant, transplant type (double or single lung), differences between donor and recipient height, and cytomegalovirus status.

Unadjusted restricted mean (SE) graft survival time at 5 years was shorter among patients who received lungs from donors in independent DCUs than hospital-based DCUs (1548 [27] days vs 1665 [50] days; difference, 117 days; *P* = .04) (Figure 2). Hazards of graft failure were also higher among lungs recovered from independent DCUs (hazard ratio [HR], 1.26; 95% CI, 1.00-1.58). Additional analyses revealed no evidence against the proportionality assumption with respect to the effect of DCU type (see eResults in Supplement 1). After stratification by transplant center and year and adjustment for extended criteria lung donor status, recipient age, sex, race and ethnicity, lung allocation score at transplant, transplant type (double vs single), difference between donor and recipient height, and cytomegalovirus status, hazards of graft failure remained significantly higher among lungs recovered from independent DCUs than hospital-based DCUs (adjusted HR, 1.85; 95% CI, 1.28–2.65) (Figure 3).

### Secondary Outcomes

Most donation process outcomes were similar between donors in independent and hospital-based DCUs. However, median (IQR) donor management time was significantly shorter among donors in independent DCUs than hospital-based DCUs (49 [8-153] hours vs 61 [10-153] hours; *P* < .001). Most secondary outcomes were similar between recipients of lungs from hospital-based vs independent DCUs, including receipt of mechanical ventilation at 72 hours and unadjusted and adjusted 1-year graft survival (eTable 3 in Supplement 1).

## Discussion

In this retrospective cohort study of national organ donor and lung transplant recipient data, overall graft survival was nearly 4 months longer among lungs recovered from donors in hospital-based DCUs. This finding, which was unexpected and refuted the study hypothesis, was consistent across analyses and robust in analyses accounting for transplant program, transplant year, and donor and recipient factors associated with graft and recipient survival.

While understanding DCU utilization and outcomes is a priority for the US Centers for Medicare & Medicaid Services^24^ and the Health Resources and Services Administration,^25^ most studies of DCUs have focused on short-term donation outcomes (including the number and type of organs recovered from donors)^8,9,26,27,28^ and donation process measures.^4,26,27,29^ This study is consistent with past work suggesting that lungs are more frequently recovered and transplanted from donors after brain death cared for in independent DCUs than acute-care hospitals,^8,9^ but is unique in its examination of longer-term lung transplant recipient outcomes and direct comparisons between independent and hospital-based DCUs.

Three potential differences between DCU types may underlie study findings: (1) donor selection, (2) donor management, and (3) relationships with transplant programs. First, observational studies of DCU processes and outcomes are inherently limited by donor selection bias; even when DCUs are available, not all deceased donors are transferred. As in past studies,^3,9,30^ individual DCUs varied in the proportion of donors transferred from area hospitals. This may reflect systematic or individual differences in available local hospital or transportation resources or donor characteristics (including clinical stability and family authorization for transfer) between areas. In this study, detectable differences between donors in each DCU type were inconsistently associated with better donation outcomes, suggesting that differences in donor acceptance practices may not be systematic. Alternatively, donor selection processes may be unique and potentially modifiable features of DCU models (eg, through expansion of clinical services to accommodate more clinically heterogeneous donors) that may be used to optimize local donation system performance.

Second, critical care nurses, respiratory therapists, and physician consultants available in hospital-based DCUs may improve graft survival over lungs recovered from independent DCUs. In this study, the only detectable management difference between recovery locations was donor management times, which were shortest in independent DCUs and longest in hospital-based DCUs. While consistent with other work focused on independent DCUs,^26,31^ interpretation of these times in isolation is difficult; shorter times may indicate better efficiency but may also miss opportunities to rehabilitate injured lungs (or other organs) before allocation and recovery or indicate a greater willingness in some DCUs to delay organ recovery until additional organs can be allocated. Further work is needed to identify modifiable differences in clinical team composition or lung management practices that may be leveraged systematically to improve lung graft quality and associated recipient survival.

Last, it is possible that transplant programs may be more willing to consider, accept, and recover lungs from donors in independent DCUs than hospital-based DCUs, either due to expectations of higher quality donor management, fewer logistical barriers (eg, predictable operating room schedules), or lower organ acquisition costs.^4,26,27,32^ While the survival model stratified by transplant program (accounting for differences in individual program practices) and year (to address changing allocation rules^33^ and the COVID-19 pandemic), the present analyses cannot account for the myriad clinical, logistical, and programmatic factors that underlie individual organ acceptance decisions.^34^ Instead, differences in donor and recipient characteristics between groups in this study likely reflect complex relationships between waitlisted patients, transplant programs, and organ availability.

While secondary comparisons of survival duration did not identify differences between independent DCUs and area hospitals, higher rates of lung acceptance from DCUs have potential benefits beyond individual recipient survival. This study further illustrates the need to distinguish between factors associated with lung acceptance and donation,^35^ primary graft dysfunction,^36^ and recipient survival.^37^ For example, in a recent study by Marklin and colleagues,^7^ a demonstrable increase in potential donors’ Pao_2_:Fio_2_ ratio after prone positioning was associated with higher lung acceptance and donation rates but no difference in recipient survival at 3 months.

In this study, long-term graft survival was unexpectedly longer among recipients of lungs recovered from hospital-based DCUs than independent DCUs or hospitals. Given the very short period of donor management relative to recipient survival, the observed relationship requires further examination. Fundamentally, understanding DCU management and outcomes needs prospective study to address selection bias inherent in decisions to transfer donors to DCUs, to characterize expected but unmeasured differences in operations and resources between DCU models, and to define measures of donor management quality and DCU-specific performance in a rapidly changing system. At present, comprehensive understanding is limited by the quality and granularity of systematically collected data on deceased organ donor management^38^ and DCU operations.

To that end, we hope that this study spurs specific research to better understand how donors are cared for in DCUs vs hospitals, how the presence of DCUs impacts organ allocation and acceptance, and how DCUs are best utilized to facilitate local and national donation system improvements. In the meantime, before transplant programs incorporate study findings into assessments of graft quality, additional work is needed to replicate results and, if observed differences in graft survival are present, to leverage those differences to maximize transplant access and recipient survival.

### Limitations

Study limitations include those inherent to the use of preexisting data. We assumed all donors were equally eligible for DCU transfer and could not account for all factors impacting decisions to transfer donors to a DCU or for donated lungs to be accepted and transplanted for individual recipients. In a US system characterized by heterogeneity of practices, resources, and outcomes among diverse hospitals,^39^ OPOs,^40^ and transplant programs,^41,42^ the impact of DCUs on individual stakeholders is also likely to vary. Furthermore, results may not be generalizable to newly opened DCUs and, given that donor management is rarely shared or transferred between organ procurement organizations, do not apply to the majority of US donor service areas without operating DCUs.^3,30^ As the study dataset lacks relevant information about the clinical conditions and management of deceased donors, we could not compare quality or intensity of donor management between groups.^5,6,43^ Similarly, we could not account for recipient care delivery variables (eg, ventilator settings) associated with short- and long-term outcomes but not captured in the study dataset.^6,37^ Additionally, some model factors, such as recipient race and ethnicity, are imprecise indicators of complex differences in social determinants of health that impact both transplant eligibility and recipient survival.^15,16,44,45^

## Conclusions

In this cohort study, among deceased donors after brain death cared for in hospital-based donor care units, transplanted lungs survived 4 months longer, on average, than lungs recovered from independent DCUs. Overall, lung donation rates were highest among donors in independent DCUs but higher among deceased organ donors after brain death cared for in DCUs vs traditional hospital settings. Further work is needed to understand and validate these differences and to identify potential interventions to increase lung transplant recipient survival and availability of transplanted lungs.

## Notes

Supplement 1. **eFigure.** Proposed Causal Diagram Describing Potential Impact of Donor Care Units on Lung Transplantation and Recipient Survival
eMethods.
**eTable 1.** Organ Donor Characteristics by Organ Recovery Site **eTable 2.** Organ Donation Processes and Outcomes by Organ Recovery Site
eResults.
**eTable 3.** Lung Transplant Recipient Outcomes by Organ Recovery Site
eReferences.

Supplement 2.
Data Sharing Statement
