# Supplementary material for: Lung Donation and Transplant Recipient Outcomes at Independent vs Hospital-Based Donor Care Units
Source: JAMA Netw Open. 2024 Jun 25;7(6):e2417107. doi: 10.1001/jamanetworkopen.2024.17107 (PMC11200140; doi:10.1001/jamanetworkopen.2024.17107)
Supplement: Supplement 1. — eFigure. Proposed Causal Diagram Describing Potential Impact of Donor Care Units on Lung Transplantation and Recipient Survival eMethods. eTable 1. Organ Donor Characteristics by Organ Recovery Site eTable 2. Organ Donation Processes and Outcomes by Organ Recovery Site eResults. eTable 3. Lung Transplant Recipient Outcomes by Organ Recovery Site eReferences. [file jamanetwopen-e2417107-s001.pdf]

Supplemental Online Content

Vail EA, Wang X, Schaubel DE, et al. Lung donation and transplant recipient outcomes at independent vs hospital-based donor care units. *JAMA Netw Open*. 2024;7(6):e2417107. doi:10.1001/jamanetworkopen.2024.17107

eFigure. Proposed Causal Diagram Describing Potential Impact of Donor Care Units on Lung Transplantation and Recipient Survival

eMethods.

eTable 1. Organ Donor Characteristics by Organ Recovery Site

eTable 2. Organ Donation Processes and Outcomes by Organ Recovery Site

eResults.

eTable 3. Lung Transplant Recipient Outcomes by Organ Recovery Site

eReferences.

This supplemental material has been provided by the authors to give readers additional information about their work.

© 2024 Vail EA et al. *JAMA Network Open.*

eFigure. Proposed Causal Diagram Describing Potential Impact of Donor Care Units on Lung Transplantation and Recipient Survival

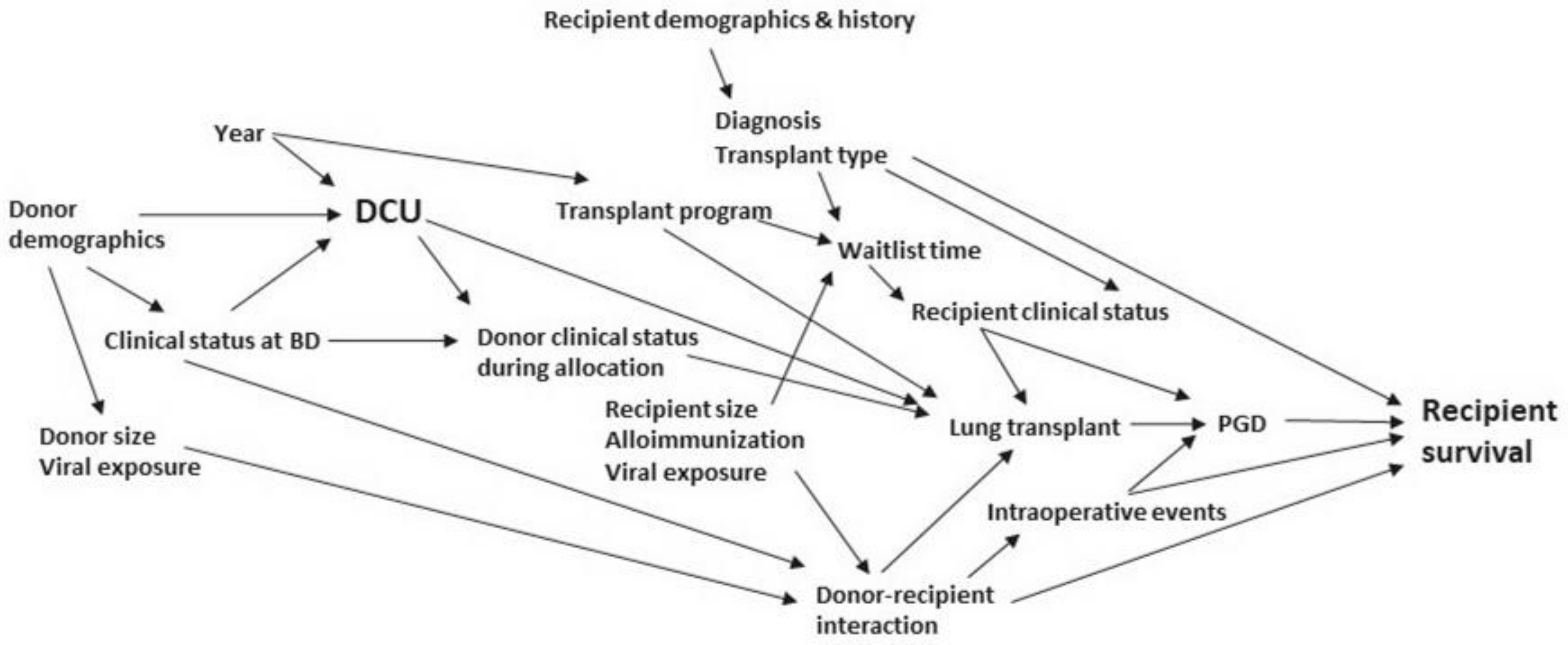

BD: brain death, DCU: donor care unit, PGD: primary graft dysfunction

© 2024 Vail EA et al. *JAMA Network Open.*

eMethods.

Secondary comparison groups

To explore whether observed differences in graft survival and donation outcomes may be attributed to the selective transfer of donors expected to donate higher quality lungs to DCUs or differences in donation processes between hospitals or DCUs, we secondarily compared donor characteristics, lung donation processes and outcomes, and recipient outcomes between donors transferred and not transferred (remaining in local hospitals) to available DCUs. To account for differences in donor populations and DCU operations between donor service areas, and because the transfer of donors between organ procurement organizations and donor service areas is rare, secondary comparisons were stratified by DCU type.

Testing of the proportional hazards assumption

After creating a multivariable Cox model, we first examined the proportionality assumption by generating baseline survival curves for each DCU type with model covariates fixed at central values (mode or median, for categorical and continuous covariates, respectively). Second, we plotted log -log curves of the survivor function (log cumulative hazards functions) from the adjusted model. Both plots were visually inspected for intersections (first plot) and constant differences over time (second plot). Third, in the adjusted Cox model, we included an interaction term between the DCU model and follow-up time.

Examining the impact of individual confounding variables

After final variable selection for the Cox model, we unpacked potential confounding mechanisms with two methods. First, we fitted sequence models that contained two predictors: the DCU model (with hospital-based DCUs as the reference group) and one additional adjustment covariate. In this set of bivariate models, we explored which selected model covariates impacted the observed relationship between DCU type and duration of lung graft survival. Second, to estimate the minimum effect size of an unmeasured confounding variable needed to nullify the observed effect of DCU model in the final Cox model, we calculated an E-value.[1]

© 2024 Vail EA et al. *JAMA Network Open.*

eTable 1. Organ Donor Characteristics by Organ Recovery Site

Type of DCU available		Hospital-based DCU			Independent DCU		
**Organ recovery location**		**DCU No. (%)**	**Hospital No. (%)**	**P value**	**DCU No. (%)**	**Hospital No. (%)**	**P value**
Donors[a]		1,466 (37.4)	2,449 (62.5)		3,683 (53.1)	3,258 (46.9)	
Demographic characteristics							
Donation year	Apr – Dec 2017	99 (6.8)	149 (6.1)	<0.001	227 (6.2)	144 (4.4)	<0.001
	2018	168 (11.5)	208 (8.5)		433 (11.8)	360 (11.0)	
	2019	195 (13.3)	289 (11.8)		625 (17.0)	807 (24.8)	
	2020	276 (18.8)	598 (24.4)		810 (22.0)	801 (24.6)	
	2021	409 (27.9)	777 (31.7)		1041 (28.3)	773 (23.7)	
	Jan – Jun 2022	319 (21.8)	428 (17.5)		547 (14.9)	373 (11.4)	
Age (years), mean ± SD		43.8 ± 15.0	42.3 ± 15.2	0.004	42.6 ± 14.7	42.8 ± 15.8	0.47
Sex[b]	Female	559 (38.1)	950 (38.8)	0.68	1477 (40.1)	1245 (38.2)	0.11
	Male	907 (61.9)	1499 (61.2)		2206 (59.9)	2013 (61.8)	
Race and ethnicity[b]	American Indian or Alaska Native	5 (0.3)	18 (0.7)	0.36	14 (0.4)	12 (0.4)	<0.001
	Asian	74 (5.0)	117 (4.8)		55 (1.5)	117 (3.6)	
	Black	271 (18.5)	403 (16.5)		764 (20.7)	529 (16.2)	
	Hispanic	302 (20.6)	548 (22.4)		405 (11.0)	722 (22.2)	
	Multiracial	22 (1.5)	32 (1.3)		12 (0.3)	33 (1.0)	
	Native Hawaiian or Other Pacific Islander	7 (0.5)	9 (0.4)		2 (0.1)	8 (0.2)	
	White	785 (53.5)	1322 (54.0)		2431 (66.0)	1837 (56.4)	
History of tobacco use		306 (20.9)	445 (18.2)	0.11	875 (23.8)	649 (19.9)	0.004
History of cocaine use		282 (19.2)	466 (19.0)	0.99	864 (23.5)	630 (19.3)	<0.001
History of other drug use		728 (49.7)	1149 (46.9)	0.28	2003 (54.4)	1499 (46.0)	<0.001
Clinical characteristics							
Donor height (cm), median (IQR)		171 (164, 178)	172 (164, 178)	0.55	173 (165, 180)	170 (164, 178)	<0.001
Body mass index (kg/m^2), median (IQR)		27.6 (23.8, 31.9)	27.5 (23.7, 32.1)	0.58	27.4 (23.7, 32.1)	27.7 (24.0, 32.6)	0.05

© 2024 Vail EA et al. *JAMA Network Open.*

eTable 1 Continued. Organ Donor Characteristics by Organ Recovery Site

**Type of DCU available**		**Hospital-based DCU**			**Independent DCU**		
**Organ recovery location**		**DCU No. (%)**	**Hospital No. (%)**	**P value**	**DCU No. (%)**	**Hospital No. (%)**	**P value**
Mechanism of death	Asphyxiation	69 (4.7)	100 (4.1)	0.38	163 (4.4)	142 (4.4)	<0.001
	Blunt injury	247 (16.8)	464 (18.9)		620 (16.8)	607 (18.6)	
	Cardiovascular	264 (18.0)	422 (17.2)		640 (17.4)	608 (18.7)	
	Death from natural causes	49 (3.3)	110 (4.5)		117 (3.2)	55 (1.7)	
	Drowning	9 (0.6)	9 (0.4)		14 (0.4)	14 (0.4)	
	Drug intoxication	200 (13.6)	303 (12.4)		693 (18.8)	419 (12.9)	
	Electrical	0 (0.0)	1 (0.0)		4 (0.1)	5 (0.2)	
	Gunshot wound	138 (9.4)	241 (9.8)		344 (9.3)	290 (8.9)	
	Intracranial hemorrhage/stroke	465 (31.7)	746 (30.5)		1005 (27.3)	1035 (31.8)	
	Other	11 (0.8)	2 (1.0)		33 (0.9)	39 (1.2)	
	Seizure	13 (0.9)	23 (0.9)		45 (1.2)	35 (1.1)	
	Stab	1 (0.1)	5 (0.2)		5 (0.1)	9 (0.3)	
Terminal PaO2:FiO2 ratio, mmHg, median (IQR)		174 (109, 412)	157 (104, 387)	0.003	190 (115, 427)	157 (109, 320)	<0.001
Pulmonary infection		1089 (74.3)	1812 (74.0)	0.84	2390 (64.9)	1981 (60.8)	<0.001

DCU: donor care unit, IQR: interquartile range, SD: standard deviation

[a]Not all cohort donors donated lungs for transplant. See manuscript Table 1 for characteristics of cohort lung donors managed in DCUs. [b]As classified by OPTN.[2]

© 2024 Vail EA et al. *JAMA Network Open.*

eTable 2. Organ Donation Processes and Outcomes by Organ Recovery Site

Type of DCU available	Hospital-based DCU			Independent DCU			Primary comparator.
Organ recovery location	DCU No. (%)	Hospital No. (%)	P- value	DCU No. (%)	Hospital No. (%)	P-value	Hospital-based vs. independent DCU[a] P-value
N donors[b]	1,466 (37.4)	2,449 (62.6)		3,683 (53.1)	3,258 (46.9)		
Donor length of stay, days, median (range)	3 (1, 9)[c]	2 (1, 368)	0.73	2 (1, 368)[c]	2.0 (1, 1099)	0.50	0.60
Donor management time,[d] hours, median (range)	61 (10, 153)	55 (8, 153)	<0.001	49 (8, 153)	52 (8, 152)	<0.001	<0.001
Number of organs transplanted, median (IQR)	3 (2,5)	3 (2, 4)	0.08	3 (2, 5)	3 (2, 4)	<0.001	0.11
At least one lung transplanted	418 (28.5)	566 (23.1)	<0.001	1233 (33.5)	862 (26.5)	<0.001	<0.001
Number of lungs transplanted, median (IQR)	0 (0, 2)	0 (0, 0)	<0.001	0 (0, 2)	0 (0, 1)	<0.001	<0.001
Machine lung perfusion[e]							
Left	29 (2.0)	37 (1.5)	0.75	87 (2.4)	56 (1.7)	0.33	0.15
Right	27 (1.8)	32 (1.3)	0.54	85 (2.3)	53 (1.6)	0.25	0.14
Total lung ischemic time,[f] hours, median (IQR)	5.5 (4.6, 6.9)	5.5 (4.5, 6.7)	0.51	5.4 (4.4, 6.6)	5.4 (4.5, 6.4)	0.36	0.16

DCU: donor care unit, IQR: Interquartile range, OPO: organ procurement organization

[a]Unadjusted comparisons. [b]Not all donors donated transplanted lungs. [c]Time from hospital admission to organ recovery, in whole or partial calendar days. Includes lengths of stay at referring hospital and DCU. [d]Time from diagnosis of brain death to organ recovery. Includes management time accrued at both the referring donor hospital and DCU. [e]Among lungs donated for transplant. [f]Among transplanted lungs. For double lung transplants, ischemic time of first implanted lung is reported.

© 2024 Vail EA et al. *JAMA Network Open.*

eResults.

Secondary comparison groups

Among organ donors managed by OPOs with operating DCUs on the date of organ recovery, 5707 remained in hospitals. DCU transfer rates were higher among areas with available independent DCUs (53.1% vs. 37.4% in areas with hospital-based DCUs, P<0.001). Donors transferred to independent DCUs had more differences from donors managed in area hospitals than donors transferred to hospital-based DCUs (vs. local hospitals), including factors variably associated with lung donation (Supplement, eTable 1).

Unadjusted short- and long-term transplant outcomes between recipients of lungs recovered in hospitals versus each DCU type were similar between groups. Significant differences in restricted mean graft survival duration were only observed between lungs recovered from donors managed in hospital-based DCUs versus area hospitals (1665 days vs. 1527, P=0.04); durations were similar between donors who underwent recovery in independent DCUs versus hospitals (1542 days vs. 1545, P=0.95). Hazards of unadjusted graft failure were not different among lungs recovered from hospital-based DCU donors vs. hospitals in those OPO areas (HR 0.78, 95% CI 0.60 – 1.01) or between independent DCUs and hospitals (HR 1.07, 95% CI 0.90 – 1.23).

Evidence of proportionality in the Cox graft survival model

In the Cox model, baseline function curves (with model covariates fixed at central values) and log (-log survivor function curves) for lungs recovered from hospital-based versus independent DCUs suggested that the proportionality assumption was satisfied. The P-value of an added interaction term (between DCU model and follow-up time) was p=0.79.

Impact of measured and unmeasured confounding variables

Sequential bivariate models demonstrated the strongest effects of transplant year and transplant center (both selected stratifying variables in the final model) on the observed relationship between the DCU type and lung graft survival. The E-value for the adjusted Cox model was 2.42 (lower limit of the 95% CI 1.67), which was larger than any estimated effect size of measured covariates in the model.

© 2024 Vail EA et al. *JAMA Network Open.*

eTable 3. Lung Transplant Recipient Outcomes by Organ Recovery Site

Type of DCU available	Hospital-based DCU			Independent DCU			Primary comparator. Hospital-based vs. independent DCU[a] P-value
Lung recovery location	DCU No. (%)	Hospital No. (%)	P-value	DCU No. (%)	Hospital No. (%)	P-value	
Short-term outcomes							
Receipt of invasive mechanical ventilation 72h after transplant	125 (30.7)	183 (33.3)	0.66	371 (29.7)	290 (33.6)	0.03	0.65
Receipt of ECMO 72h after transplant	36 (8.8)	60 (10.9)	0.30	94 (7.5)	62 (7.2)	0.25	0.50
Airway dehiscence	8 (2.0)	7 (1.3)	0.66	19 (1.5)	18 (2.1)	0.15	0.41
Acute rejection before hospital discharge	27 (6.6)	23 (4.2)	0.09	78 (6.2)	60 (7.0)	0.39	0.50
Dialysis before hospital discharge	22 (5.4)	37 (6.7)	0.66	106 (8.5)	68 (7.9)	0.63	0.09
Hospital length of stay,[b] days after transplant, median (IQR)	19 (13, 33)	19 (13, 34)	0.99	18 (13, 30)	19 (13, 30)	0.72	<0.001
One-year outcomes							
Unadjusted graft survival (%, 95% CI)[c]	89.6 (86.7 – 92.5)	88.1 (85.4 – 90.8)		88.2 (86.4 – 90.0)	89.9 (87.9 – 91.9)		
Adjusted graft survival (%, 95% CI)[d]	88.2 (86.1 – 90.4)	91.8 (89.9 – 93.7)		88.7 (87.2 – 90.2)	89.0 (87.4 – 90.7)		
Overall graft outcomes							
Restricted mean graft survival time,[a] days	1665	1527	0.04	1542	1545	0.95	0.04
Hazards of graft failure (ref. hospital) (95% CI)[a]	0.78 (0.60 – 1.01)			1.05 (0.90 – 1.23)			

CI: confidence interval, DCU: donor care unit, ECMO: extracorporeal membrane oxygenation, HR: hazard ratio, IQR: interquartile range

[a]Unadjusted. [b]Recipient lengths of stay truncated at cohort 5th and 95th percentiles (9 – 91 days) before comparison. [c]Estimated by Kaplan-Meier method, calculated at 358-365 days after transplant. [d]Estimated by Kaplan-Meier method after adjustment for all survival model covariates (extended criteria lung donor criteria, recipient age, recipient gender, lung allocation score at the time of transplant, transplant type (single vs. double), donor-recipient height mismatch, and donor-recipient CMV mismatch) and stratification by transplant center and year at 365 days after transplant.

© 2024 Vail EA et al. *JAMA Network Open.*

eReferences

1. Linden A, Mathur MB, VanderWeele TJ. Conducting sensitivity analysis for unmeasured confounding in observational studies using E-values: The evalue package. *STATA J.* 2020; 20(1): 162-175. doi:10.1177/1536867X20909696.

2. United Network for Organ Sharing. *How UNOS Collects Data*. Richmond, VA. August 1 2023. Accessed March 5 2024. Available at: https://unos.org/data/data-collection/

© 2024 Vail EA et al. *JAMA Network Open.*
