# Supplementary material for: Lung Donation and Transplant Recipient Outcomes at Independent vs Hospital-Based Donor Care Units
Source: JAMA Netw Open. 2024 Jun 25;7(6):e2417107. doi: 10.1001/jamanetworkopen.2024.17107 (PMC11200140; doi:10.1001/jamanetworkopen.2024.17107)
Supplement: Supplement 2. — Data Sharing Statement [file jamanetwopen-e2417107-s002.pdf]

Data Sharing Statement

Vail. Lung Donation and Transplant Recipient Outcomes at Independent vs Hospital-Based Donor Care Units. *JAMA Netw Open*. Published June 25, 2024.
doi:10.1001/jamanetworkopen.2024.17107

Data
Data available: No

Additional Information
Explanation for why data not available: Study data, and data dictionary, are publicly available from the Organ Procurement and Transplantation Network. Statistical code used in study analyses are available from the corresponding author upon written request.
